# Therapeutic effects of different intervention forms of human umbilical cord mesenchymal stem cells in the treatment of osteoarthritis

**DOI:** 10.3389/fcell.2023.1246504

**Published:** 2023-08-10

**Authors:** Yuelong Zhang, Huangming Zhuang, Xunshan Ren, Fuze Jiang, Panghu Zhou

**Affiliations:** Department of Orthopedics, Renmin Hospital of Wuhan University, Wuhan, China

**Keywords:** human umbilical cord mesenchymal stem cells, mesenchymal stem cells, osteoarthritis, cell therapy, extracellular vesicles

## Abstract

Osteoarthritis (OA) is a common and disabling disease. For advanced OA, surgical treatment is still the main treatment. Human umbilical cord mesenchymal stem cells (hUC-MSCs) are self-regenerative pluripotent cells, that coordinate cartilage regeneration by secreting various trophic factors, which adjust the injured tissue environment. hUC-MSCs secret extracellular vesicles and participates in OA treatment by transmitting bioactive molecules related to migration, proliferation, apoptosis, inflammatory reaction, extracellular matrix synthesis and cartilage repair. In addition, the combination of multiple substances represented by cartilage matrix and hUC-MSCs also have a significant synergistic effect on OA treatment. Because hUC-MSCs have shown considerable promise in cartilage repair, some scholars have proposed transplanting mesenchymal stem cells into damaged cartilage to delay OA progression. This article reviews the application of hUC-MSCs as a treatment for OA. With the continuous development of routine clinical applications, more reliable intervention modalities for hUC-MSCs in OA treatment will be discovered for the time to come.

## 1 Introduction

**FIGURE 1:**
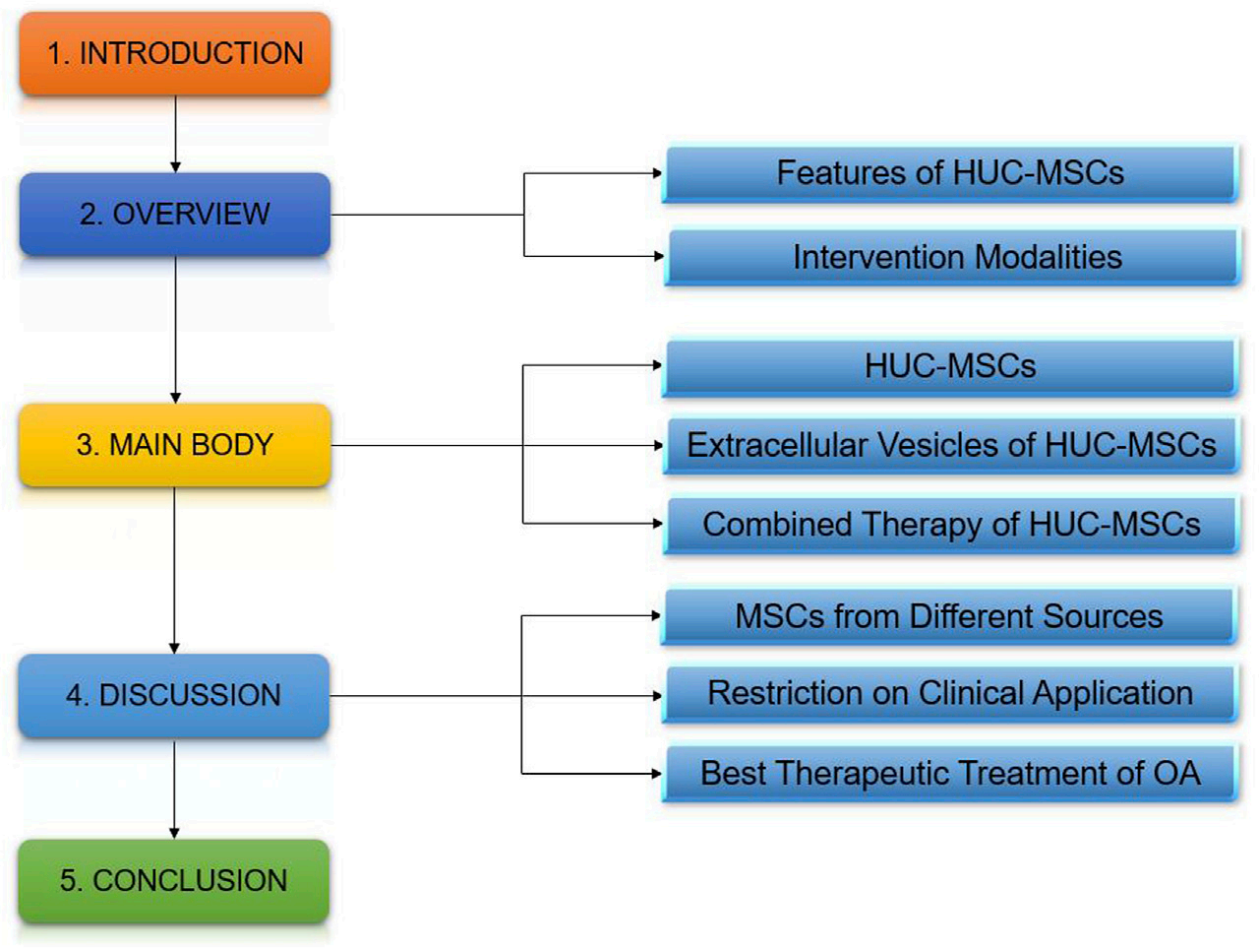
Mind map.

Osteoarthritis is a common and disabling disease which causes structural changes in articular cartilage, synovium, ligaments, capsule, and periarticular muscles (Hunter and Bierma-Zeinstra, 2019). Globally, the prevalence and incidence of OA in 2017 were 3754.2 and 181.2 cases per 100,000 persons, an increase of 9.3% and 8.2%, respectively, compared with 1990 (Safiri et al., 2020). With the increasing aging of the global population, the economic burden on the affected social and healthcare systems is increasing. OA is a total joint disease whose pathogenesis involves inflammatory, mechanical, and metabolic factors that ultimately lead to structural destruction and failure of synovial joints (Zhuang et al., 2022). The cause of the disease is a dynamic change because the imbalance between joint tissue repair and destruction rather than a passive degenerative disease or so-called wearing-off disease as commonly described. According to specific pathological processes, numerous hypotheses have proposed the etiology of arthritis, including increases in inflammatory components, mechanical overload, metabolic changes, and cellular senescence (Figure 1).

Currently, non-medicine therapies like health education and management are regarded as first-line treatments for OA (Katz et al., 2021). However, given the difficulty of this therapy being widely implemented and improving long-term patient compliance, there are still many obstacles to the treatment of OA. Joint replacement surgery is the best treatment for end-stage OA (Price et al., 2018). However, joint replacement is considered sufficiently beneficial only if the functional status of the patient’s joint is severely compromised. Current pharmacological methods to OA treatment are mainly palliative. How to improve the prognosis of OA has become the focus of drug development (Gregori et al., 2018; Deyle et al., 2020; Bandak et al., 2022). Physical therapy and intra-articular injection of glucocorticoids have been shown to be clinically beneficial in OA. However, it is uncertain whether these two therapies long-term effectiveness differ in pain relief and physical function improve (Deyle et al., 2020). Many new treatments have been proposed to reduce the symptoms of OA, such as hydrogel microspheres or platelet-rich plasma injection and targeting the subchondral bone microenvironment (Bennell et al., 2021; Hu et al., 2021; Lei et al., 2022). However, a comparison with placebo treatment has found that the magnitude of the effect of pain changes in patients is uncertain, moreover, to address uncertainties in OA efficacy, larger randomized controlled trials are needed.

Unlike previous physical or pharmacological treatments, it has been proposed that cartilage production by resident stem cell populations of bone can be induced by stimulating regenerative responses using microfracture surgery to reduce local cartilage disorders in OA (Murphy et al., 2020). This raises the question of whether we could implant some kind of allogeneic stem cells as an alternative. Nowadays, there are many stem cells from different sources are being tried for use, such as mesenchymal stem cells (MSCs). MSCs are self-regenerating pluripotent cells isolated from different human tissues. MSCs are present in almost all tissues and are involved in tissue regeneration and homeostasis (Wang et al., 2022). They have multiple characteristics, including the ability to self-renew and differentiate from multiple lineages, and can differentiate into types such as osteoblasts, adipocytes, fibroblasts, chondrocytes, and non-mesenchymal cells. Moreover, the proliferation and function of several major immune cells can be suppressed by MSCs, such as T lymphocytes, B lymphocytes, dendritic cells, and natural killer cells, and regulate immune responses (Uccelli et al., 2007; Sun et al., 2009; Liu et al., 2011). In addition, MSCs can engulf apoptotic bodies and reuse cytokines from apoptotic bodies to maintain their stem cell properties (Liu et al., 2018). Thus, MSCs have been identified as a promising source of cells for tissue regeneration and immunotherapy.

In the past, the rationale for cartilage repair using MSCs was based on their ability to differentiate into chondrocytes and replace injured cartilage (Levy et al., 2020). Increasing evidence suggests that the contribution of MSCs may lie in orchestrating the regenerative process by secreting various trophic factors that regulate the injured tissue environment or enhancing the intrinsic repair mechanisms of cartilage (Liu et al., 2021a; Perry et al., 2021). Therefore, many studies are now focused on determining the therapeutic potential of MSCs as well as their products, particularly their secreted extracellular vesicles (EVs) as a treatment for OA. EVs are a variety of nanoscale membrane vesicles actively released by cells. Vesicles of similar size can be further categorized based on their biogenesis, size, and biophysical properties (e.g., exosomes, microvesicles). It is now considered an essential carrier of cell-cell communication and circulating biomarkers for disease diagnosis and prognosis (Shao et al., 2018). Their cargo consists of several different bioactive compounds, such as proteins, enzymes, and nucleic acids, which can modulate the behavior of target cells.

Many challenges in using MSCs in clinical treatment, and clinical trials, intra-articular injection of MSCs reduces knee pain and protects cartilage. The absence of cartilage transplantation in treating OA means that another mechanism responsible probably exists for repairing cartilage. Secreted products of MSCs have anti-apoptotic, anti-inflammatory, and immunomodulatory effects and may be responsible for chondroprotective effects (Wang et al., 2020; Deng et al., 2021; Liu et al., 2022). It has been shown that the histology of cartilage destruction is improved after co-transplantation of human umbilical cord MSCs (hUC-MSCs) and hyaluronic acid (HA), while HA treatment alone is not an ideal modality (Chang et al., 2021). In addition, a major technical obstacle to mesenchymal stem cell therapy is the difficulty in isolating mesenchymal stem cells from tissue sources where low levels of mesenchymal stem cells are present, as well as the difficulty in growing these cells in sufficient quality and quantity. Some reports have revealed that platelet lysate (PL) significantly promoted the proliferation, cell cycle, and migration of hUC-MSCs. Identified beneficial effects and mechanisms of PL on MSCs and showed that PL is an effective adjuvant for MSCs in treating OA (Yan et al., 2020). These studies provide new knowledge about the clinical application of MSCs and provide a promising strategy for stem cell-based OA therapy through the combined treatment of hUC-MSCs.

In this review, we will discuss the use of hUC-MSCs and their secreted products as possible treatments for arthritic diseases, as well as the clinical benefits of multiple biological agents combined with umbilical cord MSCs.

## 2 Features of hUC-MSCs and intervention modalities

In 1995, Hillard Lazarus et al. first tested mesenchymal stem cells as a cellular agent in human subjects, 27 and have since become the most clinically studied experimental cell therapy platform in the world (Lazarus et al., 1995). In past studies, bone marrow-derived MSCs have been the most intensively studied. It has the potential for mesodermal tri-lineage differentiation and can induce differentiation into bone, cartilage, and fat under suitable conditions (Kfoury and Scadden, 2015). In addition, MSCs presented a characteristic immunophenotype: CD44, CD73, CD90, and CD105 positive as well as CD14, CD19, CD31, CD34, CD45, and HLA-DR negative (Pang et al., 2021). MSCs derived from fat, bone marrow, and umbilical cord are the most commonly investigated MSC in human clinical trials (Koliaraki et al., 2020).

One of the most significant limitations when using bone marrow MSCs (BM-MSCs) is the bone marrow aspirate process (Fan et al., 2020) as it is an invasive procedure that may carry the risk of complications. The two sources of cells are very similar: stromal cells isolated from Wharton’s jelly connective tissue and MSCs isolated from bone marrow (Lee et al., 2004). Therefore, umbilical cord MSCs (UC-MSCs) have been suggested as an alternative to BM-MSCs (Hoang et al., 2022). In the following definition of hUC-MSCs, both umbilical cord blood MSCs (UCB-MSCs) and Wharton’s jelly MSCs (WJ-MSCs) are included and are pluripotent cells (Bharti et al., 2018). In terms of cell isolation rate, extracting s

From Wharton’s jelly connective tissue is much easier. Therefore, most studies have used WJ-MSCs for the treatment of OA. Many studies have shown that the results of co-culturing WJ-MSCs with chondrocytes showed that WJ-MSCs were protective against damaged chondrocytes (Lamers et al., 2020; Liu et al., 2021a), and injection of WJ-MSCs also showed positive effects in a rat model of OA (Xing et al., 2020). Using UC-MSCs to treat OA requires an investigation of their chondrogenic potential to achieve cartilage repair. However, WJ-MSCs do not readily induce differentiation into cartilage and less frequently form hyaline cartilage tissue (Rakic et al., 2018). However, the major components of the extracellular matrix of hUC-MSCs, including collagen I and glycosaminoglycans, play an essential role in the breakdown of homeostasis and subsequent repair mechanisms caused by tissue damage (Jin et al., 2019; Koliaraki et al., 2020), and therefore remain an excellent potential for future tissue engineering applications. In summary, hUC-MSCs have been identified as having great therapeutic potential in tissue regeneration and immunotherapy.

hUC-MSCs have demonstrated some OA therapeutic effects in both animal and human studies, and researchers believe that its secreted cytokines can promote endogenous repair. Because of its high proliferative capacity, easy accessibility, and low immunogenicity, hUC-MSCs implantation has become an increasingly popular cellular therapy for the treatment of OA. Not only surgical implantation of cells, but also studies have used intra-articular injections of hUC-MSCs to treat OA, which can provide more effective pain relief and improve function compared to no cell therapy.

As mentioned earlier, Umbilical cord MSCs have shown greater potential than MSCs from other sources when used in cellular regenerative therapies. However, the use of MSCs in clinical practice still has some drawbacks that need to be improved, such as how to maintain their bioactivity, the amount of bioactive factors, and production issues. EVs are lipid-membrane-enclosed particles that fall into two main categories. Exosomes are between 40 and 150 nm in diameter and are derived from the inner nuclear plasma system; microvesicles are between 100 and 1,000 nm in diameter and are produced by shedding of the plasma membrane (Jeppesen et al., 2019). EV facilitates intercellular information transfer by transferring mRNA, microRNA, proteins and organelle cargoes to recipient cells (Liu et al., 2019a; Mathieu et al., 2019), and it can participate in apoptosis, proliferation, migration, extracellular matrix synthesis, cartilage regeneration, and inflammation management by delivering corresponding information molecules, and is considered an excellent candidate therapeutic effector for tissue regeneration and repair (Lei et al., 2021; Han et al., 2022). Many studies have demonstrated the therapeutic efficacy of EV against several cartilage-associated diseases. In recent years, researchers have found that small EVs from MSCs exhibit significant therapeutic effects on OA. Through joint cavity injection, EV can slow down OA progression and reduce cartilage damage. Compared with the direct use of hUC-MSCs, EVs are easy to produce and more likely to remain biologically active.

**FIGURE 2:**
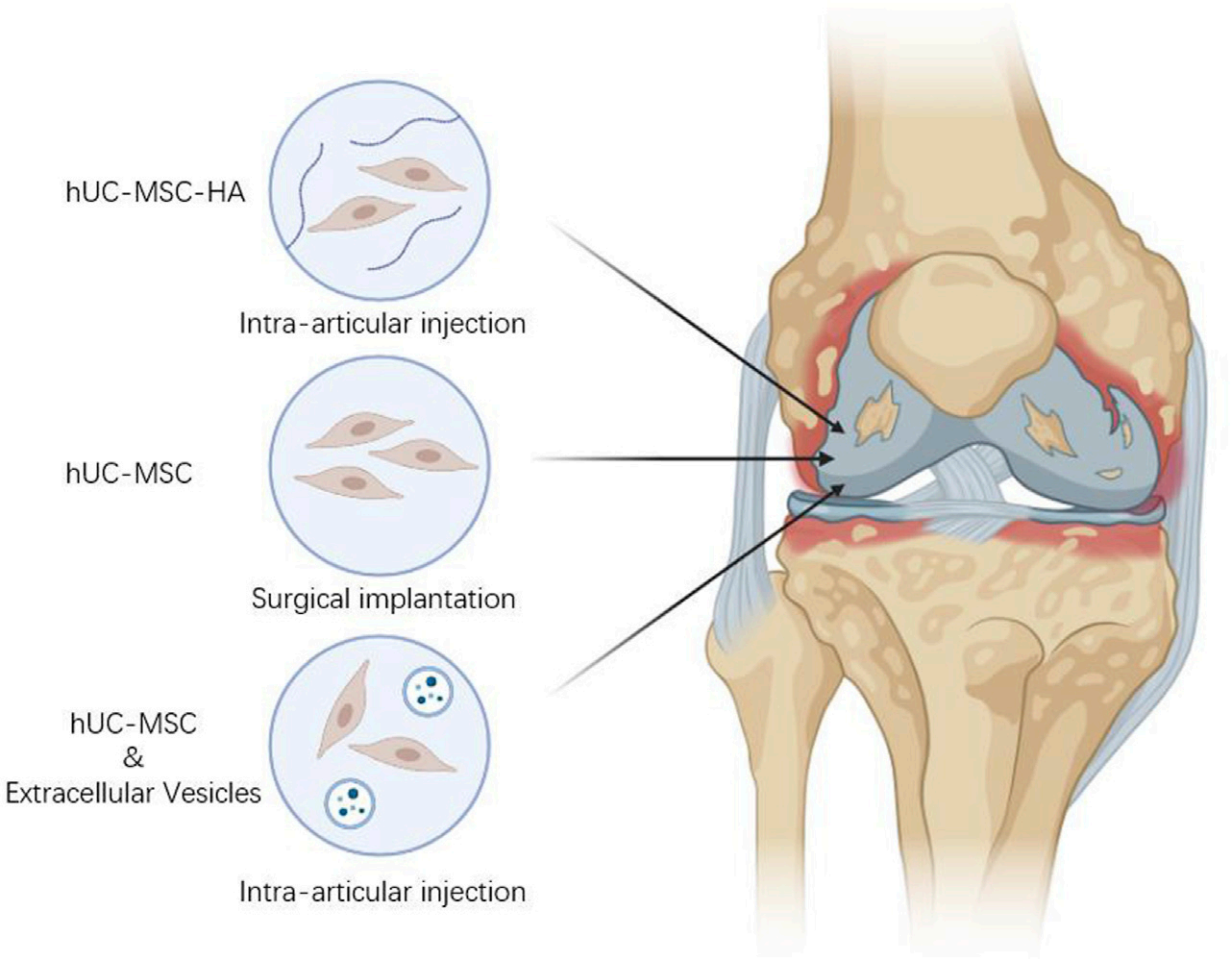
Different intervention forms of hUC-MSCs.

Evidence from *in vivo*, *in vitro*, and clinical data suggests an potential for UC-MSCs as a treatment for arthritic conditions. Several studies have shown that extracellular matrix (ECM) can be used to repair damaged parts of host tissues and restore their structure and function, and in a rabbit articular cartilage defect model, MSCs combined with cartilage extract-derived ECM regenerated damaged cartilage compared to cartilage-derived ECM alone (Yin et al., 2016). In addition, several reports have shown that PL enhanced hUC-MSCs activity, stimulated their proliferation and enhance bone tissue regeneration based on hUC-MSCs (Yan et al., 2020; Levoux et al., 2021). It is reminiscent of other substances that can be combined with hUC-MSCs for OA treatment. Combination therapy is a method of joint cavity injection or surgical implantation of hUC-MSCs together with relevant biologics for OA treatment, which produces new therapeutic effects through the interaction of stem cells with biologically active substances or the combination of hUC-MSCs with relevant substances (Figure 2).

## 3 Human umbilical cord MSCs for OA therapy

Because of their chondrogenic potential and immunomodulatory properties, and the critical role of inflammatory processes and associated articular cartilage degeneration, WJ-MSCs and human umbilical cord blood MSCs (hUCB-MSCs) are considered potential therapeutic agents for the treatment of arthritic diseases. Recent studies have been demonstrated in different animal models and clinical trials (Table 1).

**TABLE 1:** Details of the studies that used MSCs in the treatment of OA.

References	Cell types/Source	Target cells	Intervention forms	Research findings
Perry et al. (2021)	hUC-MSCs/BM-MSCs	Mouse Gdf5-expressing embryonic joint interzone cells	Intra-articular injection	Cartilage repair was significantly improved in both treatment groups
Tong et al. (2020)	hUC-MSCs	Rat chondrocytes	Intra-articular injection	hUC-MSCs have a critical role in preserving cartilage surface tissue structure, cellular function and inhibiting inflammatory responses during OA progression
Chang et al. (2022)	hUC-MSCs	Mouse chondrocytes	Intra-articular injection	In a mouse OA model, hUC-MSCs have chondrogenic potential and inhibit cartilage destruction
Zhang et al. (2021)	hUC-MSCs	Rat chondrocytes	Intra-articular injection	hUC-MSCs improve OA through prevention of cartilage degradation, restoration of chondrocyte proliferation and suppression of inflammatory responses
Yang et al. (2021)	hUCB-MSCs	Rabbit chondrocytes	Surgical implantation	After hUCB-MSCs transplantation, the overall quality of cartilage tissue was significantly improved
Lee et al. (2021)	hUC-MSCs	Human knee chondrocytes	Surgical implantation	hUCB-MSCs surgery is more effective than BMAC surgery in treating medial unicompartmental knee OA and promoting cartilage regeneration
Zhang et al. (2022a)	hUC-MSCs/BM-MSCs/AD-MSCs	Human chondrocytes	Intra-articular injection	MSCs injected intra-articularly more effective than cell-free OA therapy in reducing pain and improving function
Song et al. (2020)	hUCB-MSCs	Human knee chondrocytes	Surgical implantation	For patients with OA and medial compartment varus deformity, implantation of hUCB-MSCs with HTO is an effective treatment
Ryu et al. (2020)	hUCB-MSCs	Human knee chondrocytes	Surgical implantation	Implanting MSCs is safe and effective in repairing damaged cartilage
Yang et al. (2022)	hUC-MSCs	Human knee chondrocytes	Surgical implantation	Implantation of hUCB-MSCs was more effective than BMAC grafting in the promotion of articular cartilage regeneration
Anzillotti et al. (2022)	hUC-MSCs/BM-MSCs/AD-MSCs	Human knee chondrocytes	Intra-articular injection	Studies did not demonstrate a uniformly beneficial effect

Abbreviations: hUC-MSCs, human umbilical cord mesenchymal stem cells; BM-MSCs, bone marrow mesenchymal stem cells; OA, osteoarthritis; hUCB-MSCs, human umbilical cord blood mesenchymal stem cells; BMAC, bone marrow aspirate concentrate; AD-MSCs, adipose-derived mesenchymal stem cells; MSCs, mesenchymal stem cells; HTO, high tibial osteotomy.

The efficacy of hUC-MSCs has been further explored in different animal models. In a mouse model of focal articular surface damage in the knee joint, cartilage repair was significantly improved in the hUC-MSCs treatment group. Increased endogenous chondrocytes were detected in the repair tissue of mice treated with hUC-MSCs after 8 weeks of implantation (Perry et al., 2021). An important finding was that repeated injections of hUC-MSCs after iodoacetic acid injection significantly improved cartilage erosion and decreased OA Mankin score compared with single injections in a rat model, in addition to a significant increase in the number of superficial cells on the articular cartilage surface (Tong et al., 2020). hUC-MSCs cultured in both conventional (FBS) and exosome-free media (Exo(−)FBS) have chondrogenic potential and attenuate cartilage destruction in the mouse OA model. Besides, hUC-MSCs transplanted knees significantly decreased the expression of matrix metalloproteinases (MMP) −13 and interleukin (IL) -1β compared with OA knees (Chang et al., 2022). Some analyses have shown that intra-articular injection of hUC-MSCs delayed osteoarthritis progression by decreasing cartilage degradation, increasing Safranin-O staining, and decreasing Mankin score. In addition, hUC-MSCs significantly increasing TNF-α-induced protein 6 (TNF-α-induced protein 6) and IL-1 receptor antagonists, while decreased the expression of IL-1β and tumor necrosis factor-α (TNF-α) (Zhang et al., 2021). In a rabbit cartilage defect model, when hUCB-MSCs were transplanted into the cartilage defect, the overall mass of cartilage tissue was significantly improved. However, the expression of MMP-13 was increased in all new tissues, indicating that there may be some potential osteoarthritic changes in the new tissue (Yang et al., 2021).

The therapeutic efficacy of MSCs has already been explored in clinical trials. Transplantation of hUCB-MSCs in young patients undergoing high tibial osteotomy (HTO) is more effective than a bone marrow aspirate concentrate (BMAC) in promoting cartilage regeneration after medial unicompartmental OA of the knee (Lee et al., 2021). A meta-analysis validated the therapeutic advantages of mesenchymal stem cell therapy. For mild or moderate OA, comparative analysis of mesenchymal stem cell therapy suggests that adipose-derived MSCs (ADMSCs) and hUCB-MSCs exert better antiarthritic efficacy than BM-MSCs (Zhang et al., 2022a). However, attention needs to be paid to potential complications induced by MSCs. For patients with medial compartment OA and varus deformity, implantation of hUCB-MSCs and high tibial osteotomy (HTO) are effective treatments, and cartilage regeneration improves the clinical outcome of patients (Song et al., 2020). Comparing BMAC *versus* allogeneic hUCB-MSC implantation for cartilage defects in the knee, it could be demonstrated that BMAC or hUCB-MSC implantation is safe and effective for repairing cartilage lesions. However, more prospective studies are needed (Ryu et al., 2020). A case review study investigated 176 patients who underwent either BMAC or combined hUCB-MSC surgery for OA. Both treatments provided pain relief, improved functional scores and aspects of patient quality of life, but hUCB-MSC implantation promoted cartilage repair more effectively than BMAC (Yang et al., 2022). However, Anzillotti et al. found no evidence of a uniform beneficial effect of this therapy in patients with severe OA of the knee (Kellgren-Lawrence grade 4) treated with injectable biologics, such as platelet-rich plasma, and mesenchymal cells from bone marrow, adipose tissue, and placenta/umbilical cord (Anzillotti et al., 2022).

In conclusion, evidence from recent studies supports the therapeutic potential of hUC-MSCs in arthritic diseases, which is associated with a reduction in inflammatory molecules and a higher production of anti-inflammatory molecules. Many studies have shown that hUC-MSCs can produce large amounts of immunomodulatory factors and immunochemokines because these secretory products, rather than their ability to differentiate into chondrocytes, promote cartilage repair. Although some studies have found that hUC-MSCs have some chondrogenic capacity *in vitro*, their own poor chondrogenic capacity has been reported to be due to their inflammatory regulatory function to improve OA. endogenous repair initiated locally by stem cells is considered to be a key mechanism for tissue regeneration. Under pathological conditions such as OA, articular cartilage surface cells can be mobilised and migrate to the injured area to initiate regeneration, and thus they are crucial in endogenous repair of OA. hUC-MSCs can rapidly mobilise articular cartilage surface cells to the site of injury, initiate the repair process, and delay apoptosis of the surface cells to promote articular cartilage regeneration.

There is evidence in clinical studies that synovial inflammation plays a major role in the progression and symptoms of OA. It is considered to be the main cause of pain in OA patients, and synovial inflammation infiltrates inflammatory cells, which leads to elevated pro-inflammatory cytokines (e.g., IL-6, TNF-α, *etc.*) causing nociceptive hypersensitivity. After hUC-MSCs injection, synovial inflammation was largely eliminated. Stem cell therapy can effectively relieve patients’ pain, and hUC-MSCs implantation can be confirmed as an effective treatment in clinical trials, but more case and long-term follow-up studies are needed to determine whether it is safe and effective as well as its efficacy in patients with severe OA.

## 4 EVs of human umbilical cord MSCs for OA therapy

As mentioned earlier, hUC-MSCs showed great potential when used for OA treatment. However, improvements are still needed in clinical practice through a range of means, such as maintenance of biological activity, immune rejection, and production problems. As a result, there is increasing interest in their exosomes and small-sized EVs. In fact, these cell products have been reported to retain most of the advantages of the parental cells, including low immunogenicity, immunomodulatory properties and easier delivery of bioactive factors (Yu et al., 2014; Sane et al., 2019) (Table 2).

**TABLE 2:** Details of the studies that used EVs of MSCs in the treatment of OA.

References	Cell types/Source	Target cells	Intervention forms	Research findings
Li et al. (2022a)	hUC-MSCs	Rat chondrocytes	Intra-articular injection	hUC-MSCs-EVs slow down cartilage degradation during OA progression and can promote macrophage polarization by delivering key proteins and regulating the miRNA-mediated PI3K-Akt signaling pathway, showing strong immunomodulatory potential
Li et al. (2022b)	hUC-MSCs	Rat chondrocytes	Intra-articular injection	hUC-MSCs-Exos can reverse IL-1β-induced chondrocyte injury and modulate macrophage polarization *in vitro*
Morente-López et al. (2022)	hUC-MSCs	Rat chondrocytes	Intra-articular injection	Greater therapeutic potential exists for MSC-miR-21-derived EVs compared to MSC-miR-21, which ameliorate systemic inflammation in an *in vivo* model of OA by inactivating the ERK1/2 pathway
Zhou et al. (2022)	hUC-MSCs	Mouse knee chondrocytes	Intra-articular injection	hUC-MSCs-EVs slowed down the progression of knee OA, reduced osteophytes, increased COL2A1 and cluster protein expression, and inhibited the overexpression of ADAMTS5 and MMP13 in mice by decreasing pro-inflammatory factor secretion
Tang et al. (2021)	hUC-MSCs	Human chondrocytes	Coculture	hUC-MSCs-EVs protect cartilage from damage through many proteins associated with cartilage repair
Li et al. (2021)	hUC-MSCs	Human articular chondrocytes	Coculture	Exosomal miR-100-5p derived from hUC-MSCs inhibits cyclic exercise induced ROS generation and apoptosis in primary articular chondrocytes
Nguyen et al. (2022)	hUC-MSCs	Human chondrocytes	Coculture	TGFβ-stimulated EVs secreted by hUCMSCs upregulated the expression of chondrogenic markers (COL2 and ACAN) and downregulated the expression of fibrosis markers (COL1 and RUNX2) in chondrocytes
Zhang et al. (2022b)	hUC-MSCs	Human chondrocytes	Coculture	hUC-MSC-EVs carrying miR-181c-5p have repair effects on cartilage damage

Abbreviations: hUC-MSCs, human umbilical cord mesenchymal stem cells; hUC-MSCs-Evs, human umbilical cord mesenchymal stem cells extracellular vesicles; OA, osteoarthritis; Evs, extracellular vesicles; hUC-MSCs-Exos, human umbilical cord mesenchymal stem cells exosomes; ERK1/2, extracellular regulated protein kinases 1/2; COL2A1, collagen type 2; alpha 1 chain, ADAMTS5; ADAM, metallopeptidase with thrombospondin type 1 motif 5; MMP13, matrix metallopeptidase 13; ROS, reaction oxygen species; COL2, collagen type 2; COL1, collagen type 1.

Studies have shown that treatment with hUC-MSCs-Evs promotes polarization of M2 macrophages and expression of the anti-inflammatory cytokine IL-10 in a rat OA model, and induced M2 macrophages similarly inhibit the levels of IL-1β-induced inflammation-related factors. Therefore, hUCMSCs-EVs attenuate cartilage degradation during OA progression and mechanically can promote polarization of M2 macrophages by delivering key proteins and regulating miRNA-mediated PI3K-Akt signaling pathways, showing a strong immunoregulatory potential (Li et al., 2022a). Intra-articular injection of hUC-MSCs exosomes (hUC-MSCs-Exos) into the joints of rats with surgically induced OA prevented severe damage to knee cartilage, confirming hUC-MSCs-Exos not only promoted chondrocyte proliferation and migration but also inhibited chondrocyte apoptosis. In addition, hUC-MSCs-Exos reverses IL-1β-induced cartilage damage and modulates macrophage polarization *in vitro* (Li et al., 2022b). In a mouse OA model, it has been investigated to compare the efficacy of MSC with two treatments, miR-21 and EV, which are inhibited by lentiviral transfection. EV is the most effective therapy for reducing chemokines and cytokines in the serum of OA mice compared to MSC alone (Morente-López et al., 2022). The results showed that hUC-MSC-EVs slowed down the progression of OA in mouse knee joints by decreasing the secretion of pro-inflammatory factors, reduced osteomalacia, increased the expression of collagen type II alpha 1 (COL2A1) and cluster gel, and inhibited the overexpression of thrombochondroitin-1-motif 5 (ADAMTS5) and MMP13. *In vitro* cell line analysis showed that EVs promoted chondrocyte proliferation and migration while inhibiting apoptosis (Zhou et al., 2022).

Studies have shown that hUC-MSCs and hUC-MSCs-EVs reduced cartilage damage in animal studies. This effect was mediated by the maintenance of cartilage homeostasis, as demonstrated by the upregulation of COL II and the downregulation of MMP13 and ADAMTS5. In addition, M1 macrophage markers (leukocyte differentiation antigen CD14) were significantly decreased, while M2 macrophage markers (CD206 and IL-10) were increased in hUC-MSCs and hUC-MSCs-EV compared to untreated groups (Tang et al., 2021). Many studies have shown that microRNAs carried by exosomes are associated with various diseases. Whereas exosomes from hUC-MSCs inhibited ROS production and delayed senescence of human articular chondrocytes via the miR-100-5p/NOX4 axis, these results clarify the therapeutic role of the miR-100-5p/NOX4 axis in articular cartilage injury and provide new perspectives on clinical protocols for articular chondrocyte injury and OA (Li et al., 2021). A study confirmed that TGFβ-triggered EVs secreted by UC-MSC could upregulate the expression of chondrogenic markers (type 2 collagen and proteoglycans) and downregulate fibrotic markers (type 1 collagen and Runt-related transcription factor 2) in chondrocytes. Thus, priming of hUC-MSC with cytokines could provide selective therapeutic efficacy of EV therapy in OA and chondrocyte-related diseases (Nguyen et al., 2022). hUCMSC-EVs can be internalized by BM-MSCs and promote the proliferation, migration, and chondrogenic differentiation potential of BM-MSCs. Furthermore, miR-181c-5p could target and repress SMAD7 expression to promote bone morphogenetic protein 2- (BMP2-) -induced proliferation, migration, and chondrogenic potential in BM-MSCs (Zhang et al., 2022b).

Although experimental evidence is limited, secreted products and EVs from hUC-MSC have the potential to open a new avenue for the treatment of arthritic joint disease, potentially leading to so-called “cell-free cell therapy.”

## 5 Combined therapy of human umbilical cord mesenchymal stem cells for OA

Recently, another treatment for OA using MSCs has aroused concern. At the level of the cell and the molecule, hUC-MSC is affected by many aspects of the joint cavity, including cartilage acellular matrix (CAM), PL, and hyaluronic acid. Recently, many studies have demonstrated the therapeutic effect of the combination of corresponding joint contents and hUC-MSCs on arthritis (Table 3).

**TABLE 3:** Details of the studies that used combined therapy of hUC-MSCs in the treatment of OA.

References	Cell types/Source	Target cells	Intervention forms	Research findings
Jeon et al. (2020)	Human chondrocytes	Rabbit chondrocytes	Intra-articular injection	Therapeutic potential of hUCB-MSCs with CAM in OA via BMP6
Yan et al. (2020)	hUC-MSCs-PL	Rat chondrocytes	Intra-articular injection	The combination of PL and hUC-MSCs showed significant synergistic effects on OA
Kim et al. (2022)	hUC-MSCs- human chondrocytes	Rabbit chondrocytes	Intra-articular injection	Combined treatment with hUCB-MSCs and CAM Inj. reduces pain and effectively induces chondrocyte regeneration in OA patients
Liu et al. (2021b)	hUC-MSCs-GO	Rabbit knee chondrocytes	Intra-articular injection	The GO + hUCMSCs group was most effective in improving articular surface damage and subchondral osteoporosis
Chang et al. (2021)	hUC-MSCs-HA	Rabbit chondrocytes	Intra-articular injection	*In vitro* and *in vivo* hybrid therapy with hUC-MSCs and HA reduces cartilage tissue damage in OA
Lim et al. (2021)	hUCB-MSCs-HA	Human knee chondrocytes	Intra-articular injection	hUCB-MSC-HA implantation improves cartilage grade at arthroscopy and provides more pain relief and functional improvement for up to 5 years
Park et al. (2023)	hUCB-MSCs-HA	Human chondrocytes	Surgical implantation	After allogeneic hUCB-MSC-HA implantation with HTO, favorable clinical outcomes were achieved in all cases

Abbreviations: hUC-MSCs, human umbilical cord mesenchymal stem cells; CAM, cartilage acellular matrix; BMP6; OA, osteoarthritis; PL, platelet lysate; GO, graphene oxide, HA, hyaluronic acid; HTO, high tibial osteotomy.

CAM is a cartilage-derived ECM that promotes chondrogenesis by inducing chondrogenic differentiation of UCB-MSCs. In the rabbit anterior cruciate ligament transection (ACLT) model, CAM treatment significantly increased the expression of chondrogenic markers and bone morphogenetic protein (BMP) 6 in hUCB-MSCs. Moreover, the application of hUCB-MSCs and CAM elevated the activity of proteoglycans and type 2 collagen and promoted their production, in addition to enhancing the anti-inflammatory effects of synovial fluids (Jeon et al., 2020). Poor viability of MSCs at the transplant site often hinders MSCs-based therapeutic efficacy. Platelet lysates contain abundant growth factors that favor cell growth. The *in vitro* results showed that through the expression of target genes/proteins, PL activated the AMPK/mTOR signalling pathway through beclin1-dependent autophagy, which on the one hand shortened the cell cycle and promoted the proliferation of hUC-MSCs, and on the other hand enhanced their migration. *In vivo* studies showed that the synergistic effect of PL with hUC-MSCs had a significant therapeutic effect on OA. (Yan et al., 2020). In the goat OA model, hUCB-MSCs and CAM Inj could only improve lameness, KampL grade, and OARSI score by combined administration. In conclusion, CAM Inj promotes the differentiation of hUCB-MSCs to cartilage by multiple simultaneous administrations, thus significantly improving OA efficacy (Kim et al., 2022).

Graphene oxide (GO) has aroused much attention in the field of tissue engineering and regenerative medicine due to its unique physical, chemical, antibacterial, and biological properties (Durán et al., 2015; Halim et al., 2018). Due to its high quality physical properties, GO can collect hUC-MSCs onto its surface and immobilise them in damaged tissues. Many studies have shown that GO particle lubricants promote OA chondrocyte repair due to the lubricating properties of GO particles (Liu et al., 2019b). Moreover, GO can promote stem cell differentiation cartilage repair and improve subchondral bone (Lee et al., 2011; Lee et al., 2015). Recent studies have shown that graphene oxide particle-lubricated hUC-MSCs can promote chondrocytes, reduce joint inflammation, improve subchondral osteoporosis, and promote cartilage repair (Liu et al., 2021b). It has been reported that HA mixed with MSC in the infrapatellar fat pad may have chondrogenic potential. Compared with hUC-MSCs, gene expression of SOX9, collagen II, and proteoglycans was increased in hUC-MSC-derived chondrocytes mixed with HA compared with hUC-MSCs after chondrogenesis *in vitro*. In animal models, significant improvement in hyaline cartilage destruction after mixing hUC-MSC with HA was noticed compared to OA knees (Chang et al., 2021).

There is no optimal method to repair large full-thickness cartilage defects in elderly patients. In a randomized controlled Phase 3 clinical trial, patients were included with large full-thickness cartilage defects were treated with hUCB-MSC-HA implantation via mini-arthrotomy or microfracture. Through 5 years of observational follow-up, hUCB-MSC-HA implantation improved arthroscopic cartilage grading. In older patients with asymptomatic large full-thickness cartilage defects with or without OA, this approach is more effective in improving pain and function compared to microfracture. (Lim et al., 2021). In a retrospective study, the investigators enrolled patients who underwent high tibial osteotomy with simultaneous implantation of hUCB-MSC-HA composite for medial compartment OA and full-thickness cartilage defects of the medial femoral condyle. The results showed that good clinical efficacy and cartilage repair was achieved in all cases with allogeneic hUCB-MSC-HA composite implantation combined with HTO. These findings suggest that for patients with knee OA and full thickness cartilage defects, hUCB-MSC-HA composite implantation combined with HTO may be a promising treatment options (Park et al., 2023).

## 6 Discussion

From *in vivo*, *in vitro*, and clinical data, it is shown that UC-MSCs play an essential role in the treatment of arthritis as a representative of a variety of joint diseases, and a variety of intervention methods and mechanism studies also lay a theoretical foundation for its future development.

The therapeutic effects of hUC-MSCs in OA are mainly involved through the modulation of inflammatory response, promotion of cartilage repair, and reduction of nociceptive sensitivity. Compared with cell-free therapies, hUC-MSCs have the advantages of better anti-inflammatory effects, definite pain reduction, and better cartilage protection. The therapeutic effects exhibited by EV derived from hUC-MSCs on OA were mainly shown in the polarization of macrophages as well as the expression of anti-inflammatory factors, and EV was able to better reduce chemokines and cytokines in serum compared to hUC-MSCs alone. Not only that, hUC-MSC-EVs injected into the body were able to bind to BM-MSCs and promote the proliferation, migration and chondrogenic differentiation potential of BM-MSCs. From a clinical perspective, the use of EVs represents a new option with a similar route of administration to existing cell-free therapies, which is safer compared to surgical implantation of stem cells. However, because the bioactive components in the vesicles may have a shorter intra-articular half-life, repeated dosing may be necessary in patients with OA. In combination therapy, stem cells may have different therapeutic effects when different types of media are used. Not only do they have the same anti-inflammatory, cartilage-protecting effects as the first two, hUC-MSCs can aggregate and exert their effects more quickly with the involvement of mediators. In clinical trials, hUCB-MSCs-HA composite implantation also showed favorable therapeutic effects. In future studies, new mediators may be involved in the combination therapy, providing new ideas for OA therapy.

Although there is some substantial consensus on the beneficial effects of hUC-MSCs on diseased cartilage, there remains no clear evidence that hUC-MSCs have a considerable advantage over other MSCs from different sources in the treatment of OA. As early as 2004, Guo et al. encapsulated *in vitro* cultured autologous bone marrow mesenchymal stem cells into β-tricalcium phosphate (β-TCP) bioceramic scaffolds and implanted them into a model of articular cartilage damage. The results showed that cartilage regeneration occurred on the damaged cartilage surface and glycosaminoglycan levels were significantly increased (Guo et al., 2004). In recent years, many clinical studies have also confirmed the clinical role of bone marrow-derived MSCs. Emadedin et al. concluded that injection of autologous BM-MSCs is safe and effective in treating knee, ankle, and hip joints, with only a few patients experiencing very minor local adverse effects (Emadedin et al., 2015). Wakitani et al. demonstrated the long-term safety and efficacy of BM-MSC transplantation for cartilage repair through a follow-up study of more than 11 years (Wakitani et al., 2011).

Moreover, Koh et al. transplanted adipose-derived MSCs (ADMSCs) into knee cartilage lesions in 35 OA patients and assessed the repair efficacy using arthroscopic surgery. Patients were followed up for 2 years after ADMSC implantation without adverse effects, and the International Cartilage Repair Society (ICRS) score significantly improved (Koh et al., 2014). Freitag et al. assessed the efficacy and safety of autologous ADMSC in OA and showed significant pain relief in patients receiving ADMSCs. MRI results showed that OA patients in the ADMSCs group had decreased knee scores and cartilage regeneration in the hyaline articular cartilage of the knee joint. Two injections were more effective than one injection without severe adverse events (Freitag et al., 2019). Synovium-derived MSCs (SMSC) have a more substantial chondrogenic capacity *in vitro* (Sakaguchi et al., 2005), as well as a stable chondrogenic phenotype (Akgun et al., 2015; Kubosch et al., 2017), compared to BM-MSCs and ADMSCs. Xu et al. reported that targeted delivery of kartogenin to SMSC via engineered exosomes could promote chondrogenesis, and therefore SMSC transplantation holds promise as a new stem cell therapy for OA (Xu et al., 2021).

Recent studies have shown that various MSCs have a high safety factor in the treatment of OA and can relieve clinical symptoms or promote cartilage regeneration. However, most current studies on the treatment of OA with different MSCs lack comparison, the follow-up time of clinical studies is short, the sample size is small, there are few randomized controlled trials are involved. In addition, there is no substantial evidence to prove the advantages and disadvantages of cell types.

As we can see from previous studies, how to extract as well as preserve stem cells and their products in large and stable quantities is also an important issue to consider. Since the half-life of the biological factors contained in the secreted products in the joint environment may be very short, the effect of the cell secretion products on cartilage regeneration will only have a short-term impact and will require repeated injections. In addition, old age, large body weight, large size and moderate to severe OA are also challenges for stem cell therapy. There are no studies to suggest that stem cell therapy can replace surgical therapy for end-stage arthritis.

In the future, we can explore the best options for stem cell therapy for OA by controlling variables through additional research. These variables include the optimal therapeutic dose of MSCs in therapy, the cell type, the mode and form of implantation, the efficacy (and its durability), the method of extraction, the choice of animal model, and so on. From a clinical point of view, the possibility of using cell secretion products instead of cell therapy represents a future treatment modality, even if it is cell-based, then the cells can be transplanted in the joints to act as “factories” for the production of cellular products, while ensuring a long-term and more robust effect. In terms of industrial production, the methods for isolation and cultivation of stem cells and their products need to continue to be optimised, and not only that, but better manufacturing processes could mean advantages in terms of handling, storage and distribution. Therefore, in the future, we need to further define the advantages of these biologics, which will be more helpful in improving the existing cellular therapies in the field of OA. The treatment of cartilage repair in OA involves a wide range of disciplines, and close collaboration between multiple disciplines is needed to refine its therapeutic approach for the possible benefit of a wider range of OA patients.

## 7 Conclusion

In conclusion, hUC-MSCs and their EVs are promising candidates for the treatment of OA. Combination therapy involving MSCs can improve the viability of MSCs and enhance their biological function. This is confirmed by encouraging results from *in vitro* and *in vivo* studies as well as preliminary clinical trials. Even though EV manufacturing has advantages in easier handling, storage, and distribution, using hUC-MSCs alone and in combination has positive effects. Further studies on the treatment effects of these interventions are subsequently needed, which will help to facilitate the accuracy of what therapies are used in OA disease and provide a promising strategy for stem cell-based OA therapy.

## Abbreviations

ACLT, Anterior cruciate ligament transection; ADAMTS5, Thrombospondin-1 motifs 5; ADMSCs, Adipose-derived MSCs; BMAC, Bone marrow aspirate concentrate; BM-MSCs, Bone marrow MSCs; BMP, Bone morphogenetic protein; CAM, Cartilage acellular matrix; COL2A1, Type II collagen alpha 1; ECM, Extracellular matrix; EVs, Extracellular vesicles; GO, Graphene oxide; HA, Hyaluronic acid; HTO, High tibial osteotomy; hUCB-MSCs, Human umbilical cord blood MSCs; hUC-MSC, Human umbilical cord mesenchymal stem cell; ICRS, International Cartilage Repair Society; IL, Interleukin; MMP, Matrix metalloproteinases; MSCs, Mesenchymal stem cells; OA, Osteoarthritis; PL, Platelet lysate; SMSC, Synovium-derived MSCs; TCP, Tricalcium phosphate; TNF-α, Tumor necrosis factor-α; UCB-MSCs, Umbilical cord blood MSCs; UC-MSCs, Umbilical cord MSCs; WJ-MSCs, Wharton’s jelly MSCs.
